# Socioeconomic status and migration background as predictors of complicated lower respiratory tract infections in primary care

**DOI:** 10.1038/s43856-026-01542-5

**Published:** 2026-03-28

**Authors:** Ernst D. van Dokkum, Naomi Kraaijenbrink, Saskia Le Cessie, Martijn Sijbom, Adriënne S. van der Schoor, Leo G. Visser, Cees van Nieuwkoop, Hanneke Borgdorff

**Affiliations:** 1 Department of Public Health and Primary Care, Health Campus The Hague, Leiden University Medical Center, Leiden, The Netherlands; 2 Department of Clinical Epidemiology, Leiden University Medical Center, Leiden, The Netherlands; 3 Infectious Disease Control, Academic Collaborative Centre for Public Health Lumens, The Hague and Leiden, the Netherlands; 4 Infectious Disease Control, Public Health Service Haaglanden, The Hague, The Netherlands; 5 LUCID, Subdepartment of Infectious Diseases, Leiden University Medical Center, Leiden, The Netherlands; 6 Department of Internal Medicine, Haga Teaching Hospital, The Hague, The Netherlands

**Keywords:** Infectious diseases, Epidemiology, Epidemiology, Respiratory tract diseases

## Abstract

**Background:**

Evidence on risk factors for a complicated course of lower respiratory tract infections (LRTIs) in primary care remains limited and often consensus based. While socioeconomic status (SES) and migration background have been linked to complicated LRTIs in population-based studies, their predictive value in primary care remains unclear. Consequently, these factors are not incorporated within current guidelines, which may contribute to health inequalities. Therefore, we aimed to evaluate the added value of SES and migration background as predictive factors of a complicated course of LRTIs in primary care.

**Methods:**

Routine care data from Dutch general practices participating in the Extramural LUMC Academic Network database (Leiden-The Hague-Zoetermeer region) from 2014 to 2023, excluding COVID-19 years, was linked to sociodemographic and hospital claims data from Statistic Netherlands. Adults presenting with LRTI complaints were included (n = 145,445). Multivariable logistic regression models were constructed to predict 30-day hospitalisation or death following LRTI. Models included conventional risk factors with SES and migration background subsequently added.

**Results:**

In this study we show that after adjusting for conventional clinical factors, SES is a strong predictor of a complicated course of LRTI, whereas migration background is not. Patients in the lowest SES category have an adjusted odds ratio of 1.46 (95%CI: 1.31 – 1.62) for a complicated course compared to the highest.

**Conclusions:**

SES is a strong predictor of a complicated course of LRTI in primary care, even after adjusting for conventional risk factors. The incorporation of SES into clinical decision tools and guidelines has the potential to enhance risk-stratification of patients with LRTI in daily practice of primary care, thereby supporting more equitable care.

## Plain language summary

Lower respiratory tract infections, such as pneumonia, are common and sometimes lead to hospitalisation or death in severe cases. Currently, general practitioners use age and health conditions to assess risk, but social factors like socioeconomic status (SES) and migration background are not considered. This may lead to unequal differences in health outcomes. We analysed data from adults in Dutch general practices to see if these social factors improve predictions of severe outcomes within 30 days. We found that SES is an important predictor, even after accounting for other risk factors, while migration background had no significant effect. These findings suggest that including SES in future prediction tools and guidelines could help achieve a more equitable identification of patients at increased risk.

## Abstract (web summary)

Van Dokkum et al. examine the role of socioeconomic status and migration background as predictors of a complicated course of lower respiratory tract infections in primary care. Socioeconomic status is a strong independent predictor, whereas migration background adds no additional predictive value.

## Introduction

Lower respiratory tract infections (LRTIs) (infectious bronchitis, bronchiolitis, and pneumonia) represent the leading cause of infectious disease burden in Europe, including the Netherlands^1,2^. LRTIs can result in severe complications, such as respiratory failure and death, either directly or through related health consequences. Seasonal peaks in LRTI incidence during winter months place substantial strain on healthcare systems, a challenge that is expected to intensify due to an ageing population and the risk of emerging pandemics^3,4^.

The Dutch healthcare system is characterised by universal coverage and broad access to primary, hospital, and specialist care, ranking among the most accessible in the European Union^5,6^. Nevertheless, significant health disparities persist in various health outcomes^7,8^, as in other European countries with highly accessible care^9–11^. Within the Dutch healthcare system, general practitioners (GPs) act as gatekeepers to hospital and specialist care^12,13^. This makes primary care the central setting for managing LRTIs, which provides a unique opportunity to study LRTIs course of disease, using routinely collected, real-world primary care data.

Coughing, often indicative of an LRTI, is among the most common reasons for GP consultations in the Netherlands^14^. Severely ill patients are typically referred to hospital care the same day, while management of mild to moderate cases is guided by clinical assessment and the identification of risk factors for complications. Dutch primary care guidelines stratify patients with acute cough into three groups: patients without pneumonia and without risk factors, those without pneumonia but with risk factors of a complicated course (age >75 years or comorbidities), and those with a clinical diagnosis of pneumonia (based on symptoms such as prolonged fever, abnormal auscultation, recent pneumonia-related hospitalisation, or CRP > 100 mg/L). Dutch GPs are more likely to closely monitor and prioritise antibiotic treatment in the last two groups^15^. However, the risk factors used for stratification are largely based on expert consensus rather than robust evidence^13^.

Accurate identification of patients at risk for a complicated course of LRTIs is essential for optimising management and reducing healthcare burden. A recent systematic review by Rijk et al.^16^ identified several promising prognostic factors for complicated LRTI, including increasing age, male sex, current smoking, diabetes, history of stroke, cancer, or heart failure, hospitalisation in the previous year, the absence of influenza vaccination, current use of systemic corticosteroids, antibiotic use in the previous month, a respiratory rate >25/min, and a diagnosis of pneumonia^16^. However, the quality of evidence for these prognostic factors is limited^16^, and as these factors have not yet been combined into a validated prediction model for use in primary care, their independent predictive value remains uncertain.

Additionally, studies from the United States, United Kingdom, and the Netherlands link low socioeconomic status (SES), area-deprivation, and ethnic minorities to an increased incidence of a complicated course of LRTIs^17–23^. Nevertheless, SES and migration background have not yet been evaluated as potential predictors of complicated LRTIs, nor are they considered in current clinical guidelines, which may contribute to persisting health inequalities^13^.

In this study, we aim to evaluate the added value of SES and migration background as predictive factors of a complicated course of LRTIs in primary care. Using three multivariable regression models, we demonstrate that SES remains a strong independent predictor of a complicated course of LRTIs even after adjusting for conventional clinical risk factors, whereas migration background does not. Our findings highlight that SES captures additional vulnerability not currently accounted for in primary care risk stratification. This supports the consideration of SES in future prediction models and risk stratification approaches, which may contribute to more equitable identification of patients at increased risk.

## Methods

### Study design

We conducted a retrospective cohort study using routinely collected in-hours primary care data from general practices participating in the Extramural LUMC Academic Network (ELAN)^24,25^, covering the period between January 1, 2014, and December 31, 2023, excluding the COVID-19 period (January 1, 2020–March 31, 2022), to minimize pandemic-related bias. The ELAN-GP database^24,25^ comprises electronic health records from 1,195,698 patients that were registered with affiliated general practitioners from January 1, 2007, until December 31, 2023, in the regions of The Hague, Zoetermeer, and Leiden. These pseudonymised records were linked at the individual level to sociodemographic and hospital insurance claims data from Statistics Netherlands (SN) within the SN secure remote access environment^24,25^. The sociodemographic and hospital insurance claims data used for this study are based on data from the municipal personal records databases maintained by SN including all individuals listed in the Personal Records Database (BRP). Due to the universal healthcare coverage in the Netherlands, SN hospital insurance claims databases provide near-universal coverage of the Dutch population with data from all Dutch insurers through Vektis^26^. Prior to analysis, data were cleaned by removing duplicate records, observations without a personal identification number, and LRTI episodes within the COVID-19 period. Subsequently, databases were linked on individual level as described in Supplementary Fig. F1. All merges between datasets were validated by cross-checking identification numbers. Data from 2014 to 2022 were used to develop the prediction models and internal validation (development cohort), and data from 2023 were used for temporal external validation (validation cohort).

A flow diagram demonstrating the data linkage process with number of observations and individuals is provided in Supplementary Fig. F1.

### Participants

Patients were included if they were aged 18 years or older and presented to their GP with a new episode of an uncomplicated LRTI during the study period. LRTI episodes were identified using the following International Classification of Primary Care (ICPC) codes: acute bronchitis/bronchiolitis (R78), bronchiolitis (R78.01), influenza (R80), pneumonia (R81), SARS-CoV-2 (R83.03), and acute coughing (R05). Patients were excluded if they: 1) were hospitalised or died on the same day as the GP visit, as these episodes were already considered complicated; 2) had a new diagnosis of a major pulmonary condition (lung and respiratory tract malignancies, chronic obstructive pulmonary disease (COPD), asthma, cystic fibrosis, pulmonary embolism) within 30 days following the GP consultation, to avoid misclassification of other acute respiratory conditions as LRTI; 3) had a prior LRTI-related GP visit within six weeks, to exclude persistent symptoms; 4) were nursing home residents at the time of the LRTI episode, due to their distinct frailty profiles and risk factors. Multiple LRTI episodes per patient were allowed, provided they were separated by at least six weeks. To account for within-patient correlation due to repeated episodes, robust standard errors were calculated accounting for clustering at the patient level.

The ICPC-codes used to define major lung conditions are provided in Supplementary Table M2.

### Ethics

Informed consent from individuals in the study was waived and not obtained. In accordance with Dutch legislation, informed consent was not required for SN data per the “Law on the Central Bureau of Statistics”. Routinely collected data were pseudonymised through a trusted third party and SN. GPs informed individuals of the use of their anonymised data for research purposes and individuals could withdraw through an opt-out procedure^24,25^. For the informed consent waiver, the appropriate approval that the study was not subject to the Medical Examination Act was granted by the Medical Ethical Committee of the Leiden University Medical Centre (LUMC) under reference number 25-3023. The study protocol was approved by the Scientific Review Board of the Department of Public Health and Primary Care of the LUMC in 2025.

### Outcome measure

The primary outcome was a complicated course of LRTI, defined as all-cause hospitalisation or death within 30 days of the index GP consultation.

### Predictor variables

Predictor selection was based on a prior systematic review of Rijk et al.^16^, using the factors identified as promising prognostic factors. Of these, the following variables were selected for our analyses: age at consultation, sex, current smoking, diabetes mellitus, a history of cerebrovascular disease, a history of cancer, a history of heart failure, hospitalisation in the previous year, current use of systemic corticosteroids, antibiotic prescription in the previous month before consultation, and clinical diagnosis of pneumonia at consultation. Although a respiratory rate ≥25/min and having received an influenza vaccination in the previous year were also identified as promising prognostic factors in the review, these factors could not be included in the present study as data were not available in the SN and ELAN databases. Based on clinical relevance and prior findings, additional variables were considered: same-day antibiotic prescriptions, same-day corticosteroid prescriptions in those with pre-existing asthma or COPD (associated with risk profile, disease severity, and course of disease), and pre-existing pulmonary disease (asthma, COPD, or cystic fibrosis). In practices using point-of-care CRP testing, CRP results were also included as a sub analysis, as these guide treatment decisions and reflect disease severity.

Current smoking was defined as a binary variable (yes/no), based on the status recorded closest to the GP consultation. Smoking status was derived using structured fields and unstructured free text through text mining by using string matching^27^. Patients classified as ‘former smoker’ or missing were assumed to be non-current smokers. This assumption was validated by comparing subgroup-specific smoking prevalence trends in our cohort with national estimates from SN (Health Survey/Lifestyle Monitor; dataset 85457ENG, available from 2014 onwards. Data for adults aged ≥18 years, stratified by sex, were aligned by calendar year. Agreement in temporal trends between our cohort and national smoking estimated was assessed using Pearson correlation coefficients. Across overlapping years (2014-2019 and 2022-2023), correlations were high overall (r = 0.89), and for men (r = 0.77), and women (r = 0.96), indicating strong concordance in year-to-year changes. The absolute smoking prevalence in the cohort closely matched national estimates (24.6% vs 22.7%). However, smoking prevalence was lower than the national weighted average in the youngest age group (26.4% vs 33.9%) and higher than the national average in the oldest age group (20.4% vs 14.0%). Importantly, no interaction between smoking and age was identified in the final prediction models, suggesting that any minor age-dependent misclassification does not meaningfully affect the estimated association between smoking and the outcome. Furthermore, the strong temporal concordance suggests that our smoking status classification captures similar secular declines in smoking prevalence to national estimates.

Comorbidities were identified through Diagnosis Treatment Combination (DTC) codes (hospital care) and through ICPC-codes (primary care). A comorbidity was considered present if registered in either setting. Current corticosteroid use was defined as having an active prescription by the GP on the consultation date, excluding those initiated on the same day. Missing prescription end dates (4.6%) were imputed through multiple imputation by chained equations (MICE) and averaged across the imputations. Furthermore, antibiotic use in the previous month was defined as having a prior prescription by the GP within 30 days before the index consultation. A clinical diagnosis of pneumonia was defined as a GP diagnosis of pneumonia (ICPC R81) recorded on the day of consultation. Point-of-care CRP test results >100 mg/L were highly correlated with clinical pneumonia diagnosis. Therefore, in the CRP subgroup analysis, both variables were combined and categorised as: 1) CRP not measured/ unknown; 2) CRP 0-20 mg/L; 3) CRP 20-100 mg/L; 4) CRP > 100 mg/l or clinical diagnosis of pneumonia.

SES was defined using household-level financial prosperity, using a composite measure of the standardised annual disposable income and wealth according to the definition of SN, which categorises this composite measure into percentiles within the Dutch population^28^. SES was categorised into five groups according to quintiles in the Dutch population. Migration background was determined by an individual’s country of birth when born abroad or the parent’s country of birth when born in the Netherlands, in accordance with the SN definition^28^.

The DTC-, ATC- and ICPC-codes regarding diagnoses and medication are listed in Supplementary Tables M2 and M3. Codes used to define predictor variables and outcomes are provided in Supplementary Table M1.

### Statistics and reproducibility

The study included 185,011 LRTI episodes recorded in general practices in 145,445 patients. Each LRTI episode was considered a replicate; patients could contribute multiple episodes if separated by at least six weeks. Population characteristics were described using frequencies and percentages, medians with interquartile range (IQR) or means with standard deviation (SD). All variables were classified as dichotomous except for age, SES (ordinal), and migration background (nominal). Multivariable logistic regression models with patient-level clustering were used to develop the prediction models.

### Development of prediction models

Data from January 1, 2014, until December 31, 2022, excluding the COVID-19 period (January 1, 2020 to March 31, 2022), were used to develop three different prediction models: 1) ‘the conventional model’, including all prespecified risk factors minus SES and migration background; 2) ‘the SES model’, which added SES categories to the conventional model; 3) ‘the migration model’, which additionally included migration background. Multicollinearity was assessed using Pearson correlation coefficients; values > 0.8 indicates were considered indicative of high collinearity, but none were detected. Interaction terms were tested between key variables (age, sex, SES, migration background, and same-day antibiotic prescription) by subsequently adding interactions in multivariable logistic regression models with all prespecified risk factors. Interactions which yielded a p-value < 0.05 and increased the model fit based on likelihood ratio tests were found between same-day antibiotic prescription and pneumonia diagnosis, and between diabetes mellitus and age. Least Absolute Shrinkage and Selection Operator (LASSO) selection procedures were performed separately on the three different multivariable logistic regression models with the prespecified candidate predictors and interaction terms to select the predictive variables in each model as well as to prevent overfitting and enhance model interpretability. After the selection of predictive variables by the LASSO method, multivariable logistic regression models with the selected variables were used to develop the final predictive models to yield coefficients and probabilities. Model performance was assessed by goodness-of-fit, Brier-score, discrimination and calibration. Goodness of fit was compared between models using the Akaike Information Criterion (AIC), with differences >10 considered to provide strong support for the better-fitting model^23^. The discriminative abilities of the models were assessed using the area under the receiving operator curve (AUROC), which corresponds to the C-statistic. Calibration was evaluated and compared through the slope and intercept of calibration plots.

Observations with missing values were omitted from analyses if data on one of the included variables was missing (0.6% of cases), except for smoking status which was handled pragmatically by imputing missing values as non-current smokers, and corticosteroid use which was imputed as described above. Odds ratio’s and their corresponding 95% confidence intervals were derived from the multivariable logistic regression models with p-values < 0.05 considered significant.

We calculated observed absolute risks of a complicated LRTI course in the development cohort, stratified by the Dutch primary care clinical guideline (NHG) risk groups^15^ and SES categories. In parallel, we used the SES model to estimate the increase in mean predicted probability across the same strata to evaluate alignment between observed outcomes and model-based risk. By plotting absolute risks, we aim to enhance the clinical interpretability and relevance of found socio-economic disparities within familiar clinical categories.

Sub-analyses were performed for practices using point-of-care CRP testing, replacing the clinical pneumonia diagnosis with the combined CRP/pneumonia classification. All analyses were performed in Stata (Stata Corp. 2023. Stata Statistical Software: Release 18. College Station, TX: Stata Corp LLC.).

### Internal validation

The three models were internally validated by bootstrapping with 1000 iterations to estimate model stability. The discriminative ability, calibration and goodness of fit were subsequently compared between models.

### Temporal validation

Temporal validation was performed using data from the 2023 cohort. AUROC values of the models were compared to those in the development cohort.

## Results

### Population characteristics

The development cohort (2014-2022 without the COVID-19 period) comprised 186,094 LRTI episodes from 145,445 patients. Of these, 4,072 episodes (2.19%) progressed to a complicated LRTI. Demographic and clinical characteristics of the cohort, including its univariable association with a complicated course, are presented in Table 1.

**Table 1:** Population characteristics of the development cohort (2014-2022) and univariable associations with a complicated course of LRTI

Variable		LRTI episodes without complicated course	LRTI episodes with a complicated course	Univariable Odds Ratio (95% Confidence Interval)
Episodes n		182,022	4072	–
Sex ^a^ (% of population)	Female	108,208 (59.5)	2215 (54.4)	0.81 (0.76–0.87)
Age in years (% of population)	Median (IQR)	55 (39: 69)	73 (59: 83)	-
	18-49	74,365 (40.9)	674 (16.6)	Ref
	50-64	50,308 (27.6)	711 (17.5)	1.56 (1.40–1.73)
	65-74	30,276 (16.6)	868 (21.3)	3.16 (2.86–3.50)
	75-84	19,023 (10.5)	957 (23.5)	5.55 (5.02–6.13)
	85+	8050 (4.4)	862 (21.2)	11.81 (10.65–13.10)
Household SES ^b^ in categories (% of population)	1 (highest)	42,812 (23.5)	749 (18.4)	Ref
	2	39,361 (21.6)	680 (16.7)	0.99 (0.89–1.10)
	3	35,681 (19.6)	761 (18.7)	1.22 (1.10–1.35)
	4	34,314 (18.9)	1091 (26.8)	1.82 (1.65–2.00)
	5 (lowest)	28,821 (15.8)	741 (18.2)	1.47 (1.33–1.63)
Migration Background ^c^ (% of population)	No migration background	129,181 (71.0)	3179 (78.1)	Ref
	Middle and Eastern Europe	3358 (1.8)	21 (0.5)	0.25 (0.17–0.39)
	Turkey	9871 (5.4)	185 (4.5)	0.76 (0.66–0.88)
	Morocco	4134 (2.3)	65 (1.6)	0.64 (0.50–0.82)
	Suriname	4689 (2.6)	82 (2.0)	0.71 (0.57–0.89)
	Dutch Caribbean	8676 (4.8)	143 (3.5)	0.67 (0.57–0.79)
	Indonesia	2000 (1.1)	41 (1.0)	0.83 (0.61–1.14)
	Other Europe	7331 (4.0)	182 (4.5)	1.01 (0.87–1.17)
	Other Africa	2768 (1.5)	43 (1.1)	0.63 (0.47–0.85)
	Other Asia	7678 (4.2)	101 (2.5)	0.53 (0.44–0.65)
	Other America’s and Oceania	2333 (1.3)	30 (0.7)	0.52 (0.36–0.75)
Estimation of comorbidities (% of population)	Neoplastic disease	17,566 (9.7)	684 (16.8)	1.89 (1.74–2.05)
	Congestive heart failure	2356 (1.3)	298 (7.3)	6.02 (5.32–6.82)
	Cerebrovascular disease	3665 (2.0)	231 (5.7)	2.93 (2.55–3.36)
	Diabetes mellitus	4963 (2.7)	178 (4.4)	1.63 (1.40–1.90)
	Pulmonary disease	5923 (3.3)	262 (6.4)	2.04 (1.80–2.32)
Current smoking (% of population)		44,607 (24.5)	1019 (25.0)	1.03 (0.96–1.10)
Hospitalisation in the past year (% of population)		16,445 (9.0)	1234 (30.3)	4.38 (4.09–4.69)
Clinical diagnosis of pneumonia (% of population)		25,036 (13.8)	1746 (42.9)	4.71 (4.42–5.02)
Same-day corticosteroid prescription^d^ (% of population)		136 (0.1)	14 (0.3)	4.61 (2.66–8.01)
Current use of oral systemic corticosteroids (% of population)		2434 (1.3)	270 (6.6)	5.24 (4.60–5.97)
Antibiotic prescription <30 days before GP consultation (% of population)		6908 (3.8)	466 (11.4)	3.28 (2.97–3.62)
Same-day antibiotics prescription (% of population)		32,471 (17.8)	1018 (25.0)	1.54 (1.43–1.65)

Characteristics measured at the time of the LRTI consultation. Complications were defined as hospitalization or death within 30 days. Odds ratios are based on univariable logistic regression models using complication status as the dependent variable.

^a^Sex had 1 missing value in the uncomplicated cohort.

^b^SES: Socioeconomic status on household level, based on financial welfare (standardized income + standardized wealth of the household); 1,033 (0.6%) missing in uncomplicated cohort, 50 (1.2%) missing in the complications cohort.

^c^Migration background had 3 missing values in the uncomplicated cohort.

^d^In those with pre-existing asthma or COPD. GP: General practitioner.

The median patient age was 56 years (IQR: 39–69) and the mean was 55 years (SD: 19). Female patients comprised 59.3% of the cohort. Diabetes mellitus was present in 2.7% of the cohort, while 1.4% had a history heart failure, 9.2% had a history of neoplastic disease, and 2.1% had a history of stroke. All comorbidities were more prevalent in those who developed a complicated LRTI (Table 1). Current smoking was recorded for 24.5% of patients, and antibiotics were prescribed on the same day for 18.0% of LRTI episodes. A clinical diagnosis of pneumonia was recorded in 14.4% of cases.

Patients in the lowest (fifth) and highest (first) SES-categories comprised 15.9% and 23.4% of the cohort, respectively. Patients in the fourth and fifth categories were overrepresented among those who developed a complicated LRTI (Table 1). A migration background was recorded in 28.9% of patients; the most common were ‘other Europe’ (5.4%), Suriname (4.7%), ‘other Asia’ (4.2%), and Indonesia (4.0%), while 71.1% had no migration background.

The temporal validation cohort (2023) included 25,756 LRTI episodes, of which 585 (2.27%) progressed to a complicated LRTI. Characteristics of this cohort are detailed in Supplementary Table S2.

### Development of prediction models

Three multivariable logistic regression models were developed: 1) ‘the conventional model’, including all prespecified risk factors minus SES and migration background; 2) ‘the SES model’, which added SES categories to the conventional model; 3) ‘the migration model’, which additionally included migration background.

In the conventional model, the strongest predictors for complicated LRTI were increasing age, with an adjusted Odds Ratio (aOR) of 6.43 (95%CI: 5.71–7.23) for > 85 years, pneumonia diagnosis during GP consultation (aOR 3.10 [95%CI: 2.83–3.39]), current use of oral systemic corticosteroids (aOR 2.10 [95%CI: 1.81–2.44], hospitalisation in the past year (aOR 1.96 [95%CI: 1.80–2.13]), and diabetes mellitus (aOR 1.95 [95%CI: 1.21–3.16]) (Table 2). In the SES model, lower SES was an independent predictive factor for complicated LRTI, with the lowest SES-category demonstrating the highest odds (aOR 1.46 [95%CI: 1.31–1.62]) compared to the highest category (Table 2). In the migration model, those with a Middle and Eastern European background had an aOR of 0.55 (95%CI: 0.35–0.85) compared to those without a migration background, while other migration backgrounds showed no statistically significant associations (Table 2). Effect estimates for conventional risk factors remained stable across the three models (Table 2).

**Table 2:** Multivariable associations for a complicated course of LRTI from the conventional, SES, and migration prediction model

	Conventional model aOR (95% CI)	SES model aOR (95% CI)	Migration model aOR (95% CI)
Sex			
Male	Ref	Ref	Ref
Female	0.90 (0.84–0.96)	0.88 (0.82–0.94)	0.88 (0.83–0.94)
Age			
18–49	Ref	Ref	Ref
50–64	1.35 (1.21–1.50)	1.41 (1.27–1.58)	1.39 (1.25–1.55)
65–74	2.36 (2.12–2.63)	2.45 (2.20–2.72)	2.41 (2.16–2.68)
75–84	3.50 (3.14–3.90)	3.50 (3.14–3.91)	3.46 (3.09–3.86)
>85	6.43 (5.71–7.23)	6.36 (5.65–7.16)	6.25 (5.54–7.05)
Household SES in categories			
1 (highest)	X	–	Ref
2	X	1.11 (1.00–1.24)	1.11 (1.00–1.23)
3	X	1.27 (1.14–1.41)	1.27 (1.14–1.41)
4	X	1.36 (1.23–1.50)	1.36 (1.23–1.51)
5 (lowest)	X	1.46 (1.31–1.62)	1.47 (1.31–1.64)
Migration Background			
The Netherlands	X	X	Ref
Middle & Eastern Europe	X	X	0.55 (0.35–0.85)
Other Europe	X	X	0.88 (0.75–1.03)
Turkey	X	X	1.02 (0.78–1.32)
Morocco	X	X	0.96 (0.76–1.21)
Suriname	X	X	0.99 (0.83–1.17)
Dutch Caribbean	X	X	1.24 (0.90–1.71)
Indonesia	X	X	1.09 (0.93–1.27)
Other Africa	X	X	1.15 (0.84–1.59)
Other Asia	X	X	0.91 (0.74–1.13)
Other America’s and Oceania	X	X	1.02 (0.71–1.46)
Comorbidities			
Neoplastic disease	1.68 (1.54–1.84)	1.70 (1.56–1.86)	1.70 (1.55–1.86)
Congestive heart failure	1.73 (1.50–2.00)	1.71 (1.48–1.98)	1.72 (1.49–1.98)
Cerebrovascular disease	1.21 (1.04–1.40)	1.20 (1.03–1.39)	1.20 (1.03–1.39)
Diabetes mellitus	1.95 (1.21–3.16)	1.89 (1.17–3.05)	1.87 (1.15–3.02)
Pulmonary disease	1.20 (1.04–1.38)	1.17 (1.02–1.36)	1.17 (1.02–1.36)
Health			
Current smoking	1.08 (1.00–1.17)	1.05 (0.97–1.13)	1.05 (0.97–1.13)
Hospitalisation in the past year	1.96 (1.80–2.13)	1.92 (1.77–2.09)	1.92 (1.77–2.09)
Current use of oral systemic corticosteroids	2.10 (1.81–2.44)	2.09 (1.80–2.42)	2.09 (1.80–2.42)
Antibiotics prescribed <30 days before GP consultation	1.38 (1.22–1.55)	1.37 (1.22–1.54)	1.37 (1.22–1.54)
Degree of illness			
Clinical diagnosis of pneumonia	3.10 (2.83–3.39)	3.09 (2.83–3.38)	3.09 (2.83–3.38)
Same-day antibiotics prescription	1.09 (0.97–1.23)	1.09 (0.97–1.22)	1.09 (0.97–1.22)
Same-day corticosteroid prescription^a^	1.42 (0.75–2.70)	1.44 (0.76–2.72)	1.45 (0.77–2.73)
Interactions			
Same-day antibiotics*Pneumonia diagnosis	0.80 (0.68–0.94)	0.81 (0.69–0.95)	0.81 (0.689–0.95)
Age category 18–49* Diabetes Mellitus	Ref	Ref	Ref
Age category 50–64* Diabetes Mellitus	0.92 (0.52–1.61)	0.90 (0.51–1.58)	0.91 (0.52–1.60)
Age category 65–74* Diabetes Mellitus	0.63 (0.35–1.11)	0.62 (0.35–1.09)	0.62 (0.35–1.10)
Age category 75–84* Diabetes Mellitus	0.55 (0.31–0.99)	0.55 (0.31–0.99)	0.56 (0.31–1.00)
Age category >85* Diabetes Mellitus	0.35 (0.18–0.69)	0.36 (0.18–0.71)	0.37 (0.19–0.72)

Adjusted odds ratios are derived from the multivariable logistic regression models. Variables in all final models were selected through LASSO selection procedures, which did not exclude any prespecified variables. Patients with missing variables were excluded from analyses (*n* = 1087, 0.6%).

*CI* Confidence interval, *aOR* adjusted Odds Ratio, *GP* General practitioner.

^a^ In those with pre-existing asthma or COPD.

### Comparison of performance, calibration, and fit of prediction models

All models demonstrated acceptable-to-good discriminative ability with cross-validated AUROCs of 0.78 (95%CI: 0.77–0.79) for the conventional model, 0.78 (95%CI: 0.77–0.79) for the SES model, and 0.78 (95%CI: 0.77–0.79) for the migration model (Table 3). Each model also showed similar Brier-scores (0.020) and good calibration, indicated by calibration intercepts close to 0 and slopes near 1.0 (Table 3), consistent with minimal overfitting^29^. Calibration plots for the three models are provided in the supplementary materials Figs. F2, F3, and F4. Despite similar discrimination and calibration, the migration model showed the best overall fit, as indicated by the lowest Akaike information criterion (AIC) value (33966.76), compared to the SES model (33980.46) and the conventional model (34045.55) (Table 3). This suggests that, among the three, the migration model most effectively explains variation in the observed data^30^. However, the largest improvement in model fit occurred with the addition of socioeconomic status; the incremental gain from including migration background was comparatively modest.

**Table 3:** Model performance metrics for the conventional model, SES model and migration model for the development dataset

	Conventional model	SES model	Migration model
Cross-validated AUROC (95%CI)	0.78 (0.77–0.79)	0.78 (0.77–0.79)	0.78 (0.77–0.79)
AUROC bootstrap sample (95%CI)	0.77 (0.77–0.78)	0.78 (0.77–0.79)	0.78 (0.78–0.79)
Brier-score	0.020	0.020	0.020
Calibration intercept (95% CI)	0.01 (−0.08–0.11)	0.01 (−0.08–0.11)	0.04 (−0.06–0.13)
Calibration slope (95%CI)	1.004 (0.977–1.031)	1.004 (0.977–1.031)	1.012 (0.985–1.039)
AIC^a^	34045.55	33980.46	33966.76

^a^Akaike information criterion. CI: Confidence interval.

Internally validating the models by bootstrapping with 1000 iterations yielded similar discrimination, calibration and goodness-of-fit statistics.

### Risk assessment

To evaluate how model-predicted risk aligns with clinical guideline categories, we visualised both observed and predicted risks of a complicated course of LRTI across Dutch general practitioner society (NHG)-defined strata^15^: 1) patients without pneumonia at time of LRTI consultation and without risk factors; 2) patients without pneumonia at time of LRTI consultation but with risk factors; 3) patients with a clinical diagnosis of pneumonia at time of LRTI consultation.

**Fig. 1:**
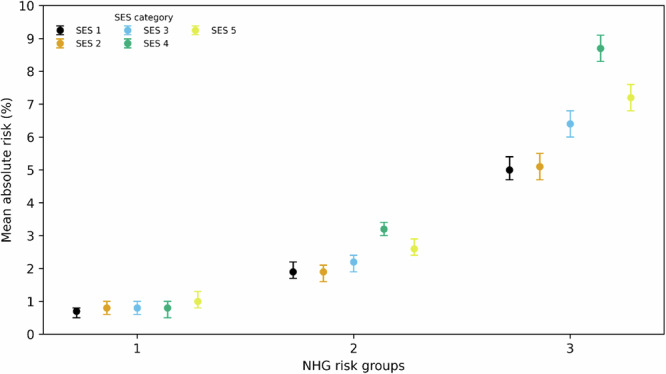
Mean observed absolute risk of a complicated course of LRTI across NHG guideline risk strata, stratified by SES. SES: socioeconomic status. NHG: Dutch general practitioner society. Note: Data are aggregated at NHG × SES level; individual-level distribution unavailable. Figure displays observed absolute risk per NHG × SES with 95% CI from binomial proportion, as recommended when raw distribution cannot be shown. Points show observed absolute risk (%) per SES category within NHG risk strata; error bars denote 95% confidence intervals (centre = mean). Estimates are based on the derivation cohort (total N = 185,011). Sample sizes (SES1–5): NHG1: 25,410; 24,246; 20,887; 16,555; 16,106. NHG2: 16,735; 15,107; 14,827; 17,285; 12,957. NHG3: 7,175; 6,157; 5,863; 6,386; 4,904. Numbers of observations per NHG guideline and SES category strata underlying the graph are also provided in Supplementary Table S11. Exact values and 95% CI bounds are provided in Supplementary Table S4. Mean absolute risk per stratum with 95% CI are derived from GLM regression with SES categories and NHG risk strata with interactions, gaussian family, identity link, robust standard errors, and Bonferroni correction for multiple comparison. NHG risk strata are defined per guidelines (1: those without risk factors or pneumonia; 2: those without pneumonia with risk factors: age >75 years, heart or lung disease, diabetes mellitus, neurological disease, liver or kidney disease, immunocompromised; 3: those with a clinical diagnosis of pneumonia which is based on clinical presentation, recent pneumonia-associated hospitalisation, or point-of-care CRP > 100 mg/L).

**Fig. 2:**
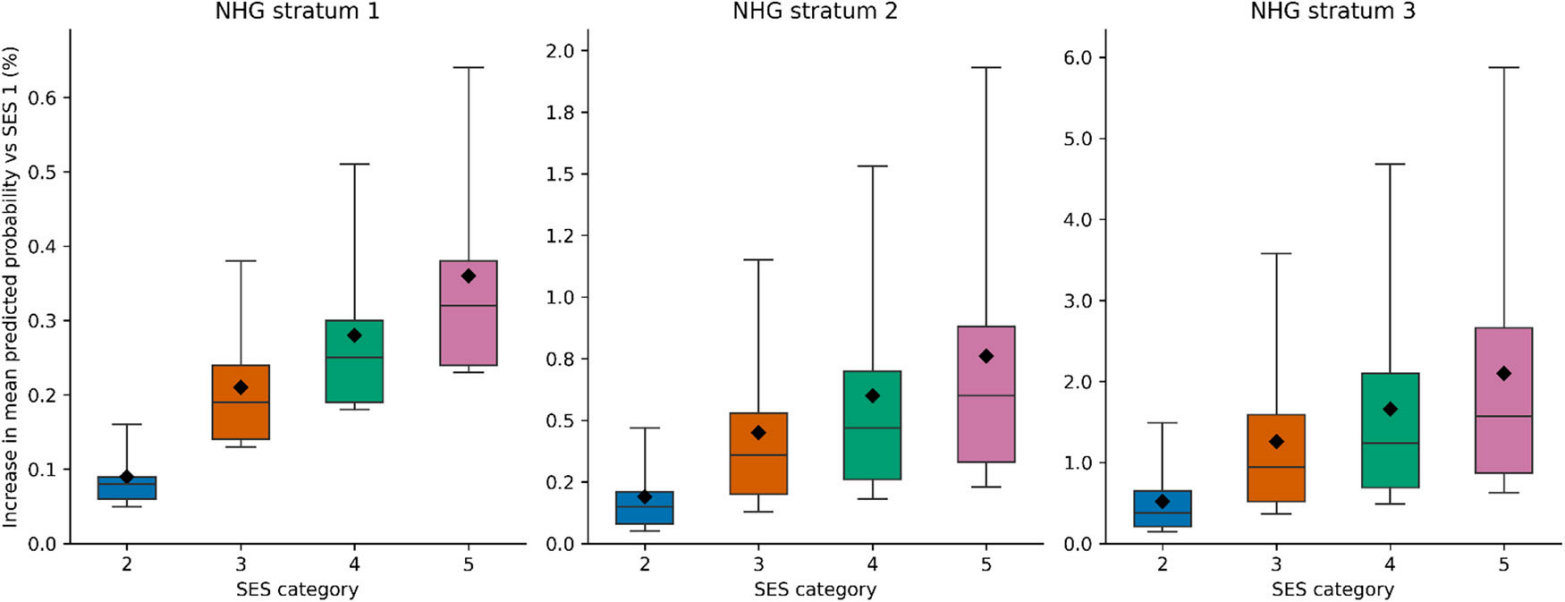
Increase in mean predicted probability of a complicated course of LRTI compared to SES category 1 across NHG risk strata, adjusted for all model covariates. SES: Socioeconomic status. NHG: Dutch general practitioner society. Marginal effects of SES on the probability of a complicated course of LRTI, by NHG risk strata. Panels show NHG strata 1–3 separately. For each SES category (2–5), boxplots represent the distribution of predicted risk differences (percentage points) relative to SES1 within the respective NHG stratum. Boxes denote median and interquartile range (Q1–Q3); whiskers indicate p5–p95 percentile bounds. Black diamonds mark the mean difference for reference. Exact values and 95%CI bounds are provided in Supplementary Table S6. Estimates are based on the derivation cohort (total N = 185,011). Sample sizes (SES1–5): NHG1: 25,410; 24,246; 20,887; 16,555; 16,106. NHG2: 16,735; 15,107; 14,827; 17,285; 12,957. NHG3: 7,175; 6,157; 5,863; 6,386; 4,904. Numbers of observations per NHG guideline and SES category strata underlying the graph are also provided in Supplementary Table S11. NHG risk strata are defined per guidelines (1: those without risk factors or pneumonia; 2: those without pneumonia with risk factors: age >75 years, heart or lung disease, diabetes mellitus, neurological disease, liver or kidney disease, immunocompromised; 3: those with a clinical diagnosis of pneumonia which is based on clinical presentation, recent pneumonia-associated hospitalisation, or point-of-care CRP > 100 mg/L).

In all NHG-defined risk strata, a gradient was observed in both observed and predicted risks across SES categories. Patients in lower SES categories consistently had higher absolute (Fig. 1) and predicted risks (Fig. 2) of a complicated course of LRTI. The difference was most pronounced in the pneumonia group, where absolute risk was 7.2% in the lowest SES category compared to 5.0% in the highest, while the predicted risk was 7.5% in the lowest SES category compared to 4.9% in the highest (Fig. 1 and Supplementary Table S4). Additionally, in the pneumonia group, the mean predicted probability of a complicated course of LRTI was 2.1% higher in the lowest SES category compared to the highest (Fig. 2).

### Temporal validation

All models showed acceptable discrimination in the 2023 validation cohort, with AUROCs slightly higher than those in the development cohort (Table 4). Differences in discrimination, calibration, and goodness of fit were similar to those observed in the development cohort.

**Table 4:** Model performance metrics for the conventional model, SES model and migration model for the validation dataset

	Conventional model	SES model	Migration model
Cross-validated AUROC (95%CI)	0.78 (0.77–0.81)	0.79 (0.77–0.81)	0.79 (0.77–0.81)
AUROC bootstrap sample (95%CI)	0.77 (0.76–0.80)	0.79 (0.76–0.81)	0.79 (0.77–0.81)
Brier-score	0.021	0.021	0.021
Calibration intercept (95% CI)	−0.04 (−0.29–0.20)	−0.06 (−0.30–0.19)	−0.03 (−0.28–0.22)
Calibration slope (95%CI)	1.005 (0.932–1.077)	1.002 (0.930–1.074)	1.010 (0.937–1.082)
AIC^a^	4882.62	4872.57	4871.49

*CI* Confidence interval.

^a^Akaike information criterion.

### Sub-analyses of patients with C-reactive protein testing

Among the 156 GP practices included in the database, 124 implemented point-of-care CRP-testing in patients presenting with LRTI, before 2023. Subgroup analyses were conducted from the moment the practice began using CRP testing. In this subgroup, CRP values were found to strongly correlate with a clinical diagnosis of pneumonia. Based on this, we defined a four-category variable combining CRP results and pneumonia diagnosis: 1) CRP not measured/unknown; 2) CRP 0-20 mg/L; 3) CRP 20-100 mg/L; 4) CRP > 100 mg/l or clinical diagnosis of pneumonia. The last category showed a significantly increased adjusted odds ratio of a complicated LRTI course (aOR 3.24, 95% CI: 1.66–6.32) compared to a CRP 0-20, consistent with findings from the main model using clinical pneumonia diagnosis alone. Associations between other covariates and complicated LRTI remained stable in this subgroup analysis and are presented in Supplementary Tables S7–S10.

## Discussion

In this large retrospective primary care cohort, we found that lower SES was an independent predictor of a complicated course of LRTI in adults, even after adjusting for conventional clinical factors such as age, comorbidities and smoking. Incorporating SES into a model with conventional risk factors significantly improved the model fit, while migration background added only marginal explanatory value. Notably, we observed a consistent SES gradient in both absolute and predicted risks across existing clinical guideline strata, suggesting that SES captures additional vulnerability not currently accounted for in primary care risk stratification.

Our findings extend previous research on LRTI prediction models^31–34^, by integrating SES and migration background into the analyses. Notably, lower SES retained its predictive value after adjusting for conventional risk factors. These results underscore that SES may serve as a practical proxy for a range of underlying risk factors relevant to infection risk and the course of disease. These may include housing quality, air pollution, vaccination uptake, working conditions, health literacy, psychological factors, access to care, and lifestyle factors such as obesity, alcohol consumption, and smoking^35–41^, which may not all be captured during GP consultations.

Although previous studies have reported associations between socioeconomic factors and complicated LRTI^17–21^, most used population-level data, thereby combining infection incidence with the risk of a complicated disease course. By contrast, our study begins at the point of GP consultation, providing more detailed insight into individual disease trajectories. We observed a clear SES gradient in both observed and predicted risk within clinical guideline-defined risk strata, particularly among patients with a clinical diagnosis of pneumonia at first consultation. Within this group, predicted risk of complication ranged from 4.9% on average in the highest SES category to 7.5% on average in the lowest, while the mean predicted probability of a complicated course of LRTI increased with 2.1% from the highest to the lowest category, highlighting a clinically relevant subpopulation at elevated risk within existing risk categories. Although these differences do not warrant changes in antibiotic treatment (as all patients in the highest-risk group are already recommended antibiotics), they may help identify patients who could benefit from closer follow-up, referral, or additional interventions (e.g., antivirals in influenza-like illness). However, the observational design of this study does not allow direct treatment recommendations and future studies are needed to explore this further.

Migration background, in contrast, showed limited predictive utility after adjustment for SES and other factors. Individuals with Middle or Eastern European backgrounds had lower adjusted odds of developing complications compared to those without a migration background. Immigrants from Middle and Eastern European countries are typically relatively healthy seasonal workers in physically demanding jobs and are, on average, younger than 35 years^42,43^. Additionally, their stay in the Netherlands is often short-term, usually less than five years^44^. These factors may contribute to the “healthy immigrant effect”, a phenomenon in which younger migrant populations, often migrant workers, tend to arrive in better overall health than the native population^45^. However, this population of migrant workers is also known to experience reduced access to healthcare services^43,46^. This combination of better baseline health and healthcare access barriers may possibly contribute to lower hospitalisation rates. This should not be interpreted as evidence that these individuals require less clinical attention; rather, it may reflect under-recognition of complications due to access barriers. As such, we do not recommend modifying clinical strategies based on these findings.

In contrast to our findings, studies from the United States linked ethnic minorities to influenza-associated hospitalisation and mortality incidence^22,23^. The discrepancy in these findings may partly be explained by major differences in healthcare systems, such as the absence or presence of universal coverage and mandatory basic insurance. Furthermore, studies on the effect of ethnicity and migration background not accounting for SES may have overlooked the confounding effect of SES and attributed mostly SES-driven associations to ethnicity or migration background. Our findings underscore the importance of separating structural determinants such as SES from ethnic or migration-based categories when developing prediction models.

Our model performed well in internal and temporal validation, achieving higher discrimination (AUROC/C-statistic 0.78) than previously published primary care prediction models for LRTIs (with C-statistics ranging from 0.63 to 0.74)^31,32,34^. While we lacked some clinical metrics such as oxygen saturation, blood pressure, and respiratory rate^32,34^, we accounted for disease severity through proxies including pneumonia diagnosis, CRP results in our sub analysis, and medication prescription patterns. The current quality of evidence supporting many of the previously identified promising prognostic factors has been low to very low^16^. The relative robustness of conventional risk factors for complicated LRTIs in our models supports and extends earlier evidence^16,31–33,47^, while demonstrating the added value of incorporating SES.

In 2020, only 0.2% of the Dutch population reported unmet needs for medical care, underscoring that access to care is generally not a significant barrier within the Dutch healthcare system^6^. Nevertheless, our findings demonstrate that health inequalities persist in the context of complicated LRTIs, similar to other, health outcomes^7,8,10,11^. This reinforces the notion that disparities are driven not solely by access barriers, but also by broader social determinants. Given the similar population composition and healthcare infrastructure of many European countries, our results likely generalise beyond the Dutch context. Indeed, similar patterns of social deprivation and poor LRTI outcomes have been reported in the United Kingdom (UK)^20^. The applicability of SES as a predictor, with the definition used in the present study, may vary across healthcare contexts. To enable broader applicability, future research should explore context-specific adaptations or identify pragmatic proxies for SES such as deprivation indices or postal code to facilitate its incorporation into risk stratification across diverse healthcare systems.

Strengths of our study include its large sample size, representative primary care setting, and linkage of real-world clinical data to detailed sociodemographic data and hospital outcomes. The gatekeeper role of Dutch GPs allowed for unique insights into LRTI course of disease from first presentation. Moreover, including conventional risk factors, SES, and migration background allowed for a more nuanced understanding of their relative contributions. Limitations include the observational nature of the study, precluding causal inferences. As this study is based on routinely collected primary care data, some residual confounding may persist and differences in diagnostic behaviour cannot be fully excluded. Influenza vaccination status was unavailable, but as influenza represents only a subset of LRTIs, its impact on our findings is likely limited. Misclassification and missing data are inherent to routine data; however, linkage with Statistics Netherlands helped to minimise these issues. Missing values regarding smoking status were imputed as non-current smokers, which may introduce differential misclassification. However, as shown in our validation against national estimates, any resulting bias is likely minimal. Moreover, annual income may fluctuate over time with potential major reductions in income due to severe illness or death in the same year. To minimise this bias, we used household financial prosperity from the preceding year rather than the year of observation to enhance stability and validity. Additionally, as we were only able to internally and temporally validate the prediction models it is uncertain how these models would perform in other parts of the Netherlands, in other countries, or in other (out-of-office) healthcare settings. Finally, the model was designed to estimate risk under current care rather than directly guide clinical decision-making; further work is needed to translate it into actionable tools.

In summary, our study establishes low SES as a significant and independent predictor of a complicated course of LRTIs in Dutch primary care, even after accounting for conventional risk factors, while demonstrating that migration background is not. Additionally, we observed a consistent SES gradient in both absolute and predicted risks across existing primary care guideline strata, highlighting that SES captures additional vulnerability not currently accounted for in primary care risk stratification. These findings support the consideration of SES in future prediction models and risk stratification approaches, which may contribute to more equitable identification of patients at increased risk. At the same time, the association between SES and a complicated disease course likely operates through a range of intermediate factors, and further research is needed to clarify the mechanisms underlying this relationship. Beyond individual risk stratification, our findings also support the design of tailored public health strategies, such as focused vaccination campaigns in low-SES neighbourhoods where uptake is typically lower. Leveraging routinely available indicators such as postcode-based deprivation indices and integration within electronic health records could facilitate this integration in practice. However, future studies should evaluate feasibility, acceptability and impact on patient outcomes. Overall this study provides a foundation for more socially responsive risk stratification, offering a concrete step toward recognizing and addressing SES-linked vulnerability in patients with LRTIs.

## Supplementary information

Supplementary Material

Supplementary Data 1

## Data Availability

The data that support the findings of this study may be obtained from a third party and cannot be shared publicly due to the current Dutch legislation for data protection of Statistics Netherlands data. Data is stored by Statistic Netherlands. Under certain conditions, the dataset and additional microdata are accessible for statistical and scientific research and must be directly requested from Statistics Netherlands (microdata@cbs.nl). Source data for Fig. 1 and Fig. 2 are provided in Supplementary Data 1.

## Code availability

All code required to reproduce the results of this study are disclosed through a repository on GitHub: https://github.com/elan-dcc/VanDokkum26^48^. All analyses were performed in Stata (Stata Corp 2023. Stata Statistical Software: Release 18. College Station, Texas, USA: Stata Corp LLC).
