# Supplementary material for: Socioeconomic status and migration background as predictors of complicated lower respiratory tract infections in primary care
Source: Commun Med (Lond). 2026 Mar 28;6:297. doi: 10.1038/s43856-026-01542-5 (PMC13194730; doi:10.1038/s43856-026-01542-5)
Supplement: Supplementary file 1 — Supplementary Material [file 43856_2026_1542_MOESM1_ESM.pdf]

Supplementary Appendix: The predictive value of socioeconomic status and migration background for complicated lower respiratory tract infections in primary care

M-Tables

Table M1 – Definitions of variables used in the study

Table M2 – DTC- and ICPC-codes used for definition of variables and LRTIs

Table M3 – Drugs by ATC4-codes, used to help define Diabetes Mellitus, Immunosuppression, Antibiotics and corticosteroids.

S-Tables

Table S1 – Population characteristics stratified by antibiotic prescription on the same day

Table S2 – Population characteristics of the validation dataset

Table S3 – Difference in deviance and deviance ration between models

Table S4 – Absolute risk and predicted risk of a patient's risk of developing a complicated LRTI classified into risk-groups according to NHG guidelines, stratified by SES-quintiles in derivation dataset

Table S5 – Absolute risk and predicted risk of a patient's risk of developing a complicated LRTI classified into risk-groups according to NHG guidelines stratified by SES-quintiles in validation dataset

Table S6 – Increase in mean predicted probability of a complicated course of LRTI across NHG risk strata, adjusted for all model covariates

Table S7 – Multivariable logistic regression sub analyses with CRP added in the model, only practices where a CRP was possible are included

Table S8 – Difference in discrimination between models with CRP-POC test integrated

Table S9 – Difference in calibration between models with CRP-POC test integrated

Table S10 – Difference in deviance and deviance ratio between models with CRP-POC test integrated

Table S11 – Number of observations per NHG guideline and SES category strata in the derivation cohort

F-Figures

Figure F1 – Flow diagram of data linkage process with number of observations and individuals

Figure F2 – Calibration plot conventional model

Figure F3 – Calibration plot SES model

Figure F4 – Calibration plot migration model

M-References

Table M1 – Definitions of variables used in the study

Variable	Definition	Dataset	Code
**A complicated course of LRTI (outcome)**	All-cause hospitalization or death within 30 days of index GP consultation.	ELAN: Episodes → for GP consultations with ICPC codes<br><br>SN: MSZPRESTATIESVEKTTAB (2016-2023) MSZSUBTRAJECTENTAB (2014-2015) → Hospitalisations<br><br>GBAOVERLIJDEN2023TABV1 → death within 30 days after GP consultation	ELAN: ICPC and dBegindatum_Copy<br><br>SN: Hospitalisation → → VEKTMSZSettingZPK = 3 from 2016 to 2023 MSZSTRSettingZPK = 3 in 2014 and 2015<br><br>Death → GBAOVERLIJDENSDATUM
**Age**	Age at GP consultation	SN: GBAPERSOONTAB (GBAPERSOON2023TABV1)	birthday=mdy(GBAGEBOORTEDAG, GBAGEBOORTEMAAND, GBAGEBOORTEJAAR)<br><br>age = (consultation date - birthday)/365.25
**Sex**	Sex assigned at birth	SN: GBAPERSOONTAB (GBAPERSOON2023TABV1)	GBAGESLACHT
**Household Socioeconomic Status (SES)**	SES was defined at the household level as financial prosperity using a composite measure of the household's standardised annual disposable income and wealth [1]. SN provided household financial prosperity in percentiles (ranging from 1–100) within the entire Dutch population. Household wealth encompasses the total value of financial assets, extracting any outstanding liabilities [1]. To ensure accuracy, household financial prosperity from the previous year rather than the current year was used, accounting for potential major reductions in annual income due to severe illness or death. Additionally, if SES data were missing in the year prior to the observation, the available SES data closest to the particular year were used.<br><br>SES was divided into 5 categories for analyses ranked from the wealthiest 20% of the Dutch population (Cat 1) to the 20% poorest of the Dutch population (Cat 5).	SN: VEHTAB (yearly dataset for separate years)	VEHP100WELVAART
**Migration background**	The term 'migration background' was used for foreign-born individuals and individuals born in the Netherlands with one or	SN: GBAPERSOONTAB and LANDAKTUEELREFV12	GBAHERKOMSTGROEPERING<br>landtiendeling & LANDAKTUEEL12

	two parents born abroad. Migration background was determined by an individual’s country of birth when born abroad or the parent’s country of birth when the individual was born in The Netherlands [1]. If both parents were born abroad, migration background was determined based on the mother’s country of birth [1]. When an individual and their mother were born in the Netherlands, migration background was defined by the country of birth of the non-Dutch father [1].		
**Comorbidities**	Comorbidities were identified through ICPC-codes registered at the GP and additional DTC registrations and medication claims. These were classified into larger categories based on the Pneumonia Severity Index (PSI) categories as defined by Fine et al.: neoplastic disease, congestive heart failure, cerebrovascular disease [2]. In addition, the presence of pulmonary disease and diabetes mellitus was assessed though DTC-claims and ICPC-codes.<br><br>All DTC-codes and ATC-codes that were used to define comorbidities can be found in Supplementary Table M2 and M3.	SN: MSZPRESTATIESVEKTTAB (2016 - 2023) and MSZSUBTRAJECTENTAB (2014 - 2015)<br><br>ELAN: Episodes	SN: VEKTMSZSpecialismeDiagnoseCombin (2016-2023) and MSZSTRSpecialismeDiagnoseCombin (2014 - 2015)<br><br>ELAN: ICPC
**Current smoking**	Current smoking was defined as a binary variable (yes/no), based on the status recorded closest to the GP consultation. Smoking status was derived using structured fields and unstructured free-text through text mining by using string matching. Patients classified as ‘former smoker’ or missing were assumed to be non-current smokers.	ELAN: JRN_Roken	Roken
**Hospitalisation in the previous year**	As a previous hospitalisation might also increase the risk of a future complicated LRTI, a covariate was included on whether a patient was hospitalised in the past year. This was defined as an individual who had been hospitalised within the 365 days prior to the observation.	SN: MSZPRESTATIESVEKTTAB (2016 - 2023) and MSZSUBTRAJECTENTAB (2014 - 2015)	VEKTMSZSettingZPK = 3 from 2016 to 2023<br><br>MSZSTRSettingZPK = 3 in 2014 and 2015

**Clinical diagnosis of pneumonia**	Clinical diagnosis with pneumonia by GP recorded at the index GP consultation.	ELAN: Episodes	ICPC → R81
**Same-day corticosteroid prescription**	New corticosteroid prescription by the GP on index LRTI GP consultation. Only for those with pre-existing Asthma or COPD. Also defined as a Asthma/COPD exacerbation.	ELAN: Medication	dATC
**Current use of oral systemic corticosteroids**	First corticosteroids prescription date before index GP consultation and corresponding prescription end date after index GP consultation	ELAN: Medication	dATC
**Same-day antibiotics prescription**	Antibiotics prescription by GP on same day as index GP consultation.	ELAN: Medication	dATC
**Antibiotic prescription <30 days before GP consultation**	Antibiotics prescription by GP within 30 days prior to index GP consultation	ELAN: Medication	dATC
**CRP**	CRP point of care test outcome value in GP practices where CRP machine was present	ELAN: LAB	dWCIANnummer → 3755

Table M2 – DTC- and ICPC-codes for definition of variables and LRTIs

**Variable**	**DTC-codes**	**ICPC-codes**
**Lower respiratory tract infection**	-	R05, R78, R78.01, R80, R81, R83.03
**Neoplastic disease [2]**	0302#21, 0302#60, 0302#61, 0302#62, 0302#63, 0302#64, 0302#65, 0302#66, 0302#67, 0302#68, 0302#69, 0302#72, 0302#84, 0303#303, 0303#306, 0303#318, 0303#319, 0303#331, 0303#332, 0303#333, 0303#334, 0303#335, 0303#346, 0303#347, 0303#349, 0303#350, 0303#352, 0303#353, 0303#357, 0303#358, 0303#360, 0303#363, 0303#367, 0303#370, 0305#1110, 0305#1140, 0305#1150, 0306#10, 0306#16, 0306#20, 0306#30, 0306#40, 0306#45, 0306#48, 0306#50, 0306#60, 0306#69, 0306#70, 0306#78, 0306#84, 0306#92, 0307#M11, 0307#M12 0307#M13 0307#M14, 0307#M15, 0307#M16, 0307#M99, 0313#214, 0313#264, 0313#621, 0313#622, 0313#623, 0313#624, 0313#629, 0313#751, 0313#752, 0313#753, 0313#754, 0313#756, 0313#757, 0313#761, 0313#771, 0313#801, 0313#802, 0313#811, 0313#821, 0313#822, 0313#823, 0313#831, 0313#832, 0313#833, 0313#834, 0313#839, 0313#841, 0313#842, 0313#843, 0313#899, 0313#904, 0313#914, 0313#964, 0313#979, 0318#307, 0318#407, 0318#408, 0318#610, 0318#712, 0318#735, 0322#1303, 0322#1304, 0322#1305, 0322#1306, 0322#1308, 0330#202, 0330#203, 0330#213, 0330#223, 0330#233, 0330#242, 0330#243.	A79, B72, B73, B74, D74, D75, D76, D77, F74.01, H75.01, K72.01, L71 (not L71.02), N74, R84, R85, S77 (not S77.01), T71, U75, U76, U77, W72, X75, X76, X77, Y77, Y78
**Liver disease [2]**	0313#463, 0313#941 0313#942, 0313#943, 0313#944, 0313#945,	D72, D97

	0313#946, 0318#701, 0318#705, 0318#707, 0318#708, 0318#709, 0318#713, 0318#718.	
**Congestive heart failure [2]**	0313#107, 0320#301, 0320#302, 0335#262.	K77
**Cerebrovascular disease [2]**	0313#121, 0330#1102, 0330#1111, 0330#1112, 0335#263.	K89, K90
**Chronic renal disease [2]**	0313#324, 0313#325, 0313#331, 0313#332, 0313#336, 0313#339.	U99.01
**Pulmonary disease (other)**	0303#309, 0313#601, 0322#1201, 0322#1241, 0322#1403, 0335#272.	R91, R95, R96, T99.10
**Neurologic disease**	0330#501, 0330#522, 0330#531, 0330#911, 0330#999, 0335#252.	N86, N87, N99.01, N99.02, N99.03
**Diabetes Mellitus**	0313#221, 0313#222, 0313#223, 0318#902, 0335#222	T90
**Immunocompromised**	0303#551, 0303#553, 0303#554, 0303#555, 0303#557, 0303#559, 0303#560, 0303#561, 0303#562, 0303#563, 0313#70, 0313#72, 0313#73, 0313#74, 0313#76, 0313#78, 0313#79, 0313#81, 0313#82, 0313#83, 0318#761 0318#763 0318#764, 0318#766, 0318#767, 0318#768, 0328#2910, 0328#2920, 0328#2930, 0303#325, 0303#326, 0305#1394, 0313#501, 0313#503, 0313#512, 0313#515, 0313#521, 0313#522, 0313#523, 0313#524, 0313#525, 0313#526, 0313#527, 0313#922, 0313#923, 0318#601, 0318#602, 0324#101, 0324#102, 0324#114, 0324#201, 0324#202, 0324#301, 0324#302, 0324#304, 0324#305, 0324#306, 0324#307, 0324#311, 0324#312, 0324#313, 0324#315, 0324#316, 0324#317, 0324#318, 0324#319, 0313#461, 0313#462.	A87.02, B72, B73, B90, T99.01, B74.01<br>Rest was defined through medication use (such as immunosuppressives for colitis ulcerosa and rheumatoid arthritis)
**Other cardiovascular disease**	0303#412 0303#418, 0303#419, 0303#420, 0313#124, 0313#133, 0320#202 0320#203, 0320#204, 0320#205, 0320#601, 0320#801, 0320#802, 0320#803, 0320#804, 0328#2220, 0328#2320, 0328#2400, 0328#2415, 0328#2425, 0328#2470, 0328#2550, 0328#2555, 0328#2560, 0328#2570, 0328#2585, 0328#2630, 0328#2635, 0328#2640, 0328#2645, 0328#2650, 0328#2655, 0328#2665, 0328#2720, 0328#2740, 0328#2770, 0328#2785, 0328#2940, 0328#3210, 0328#3310	K74, K75, K76, K78, K84, K91, K92.01, K99.01
**Major pulmonary conditions used for exclusion criteria (**lung and respiratory tract malignancies, COPD, Asthma, cystic fibrosis, pulmonary embolism**)**	-	R84, R85, R95, R96, T99.10, K93

Table M3 – Drugs by ATC4-codes, used to help define Diabetes Mellitus, Immunosuppression, Antibiotics and corticosteroids.

ATC4-code [3]	Name
**Diabetes Mellitus Medication**	
A10A	Insulins and analogues
A10B	Blood glucose lowering drugs, excluding insulins
**Immunosuppressives**	
L04A	Immunosuppressive drugs
L01B	Antimetabolites
L01X	Other antineoplastic agents
H02A	Corticosteroids for systemic use, plain
**Antibiotics**	
J01A	Tetracyclines
J01B	Amphenicols
J01C	Beta-lactam antibacterials, penicillin's
J01D	Other beta-lactam antibacterials
J01E	Sulphonamides and Trimethoprim
J01F	Macrolides, Lincosamides and Streptogramins
J01G	Aminoglycoside antibacterials
J01M	Quinolone antibacterials
J01R	Combination of antibacterials
J01X	Other antibacterials

Table S1: Population Characteristics stratified by antibiotics prescription on the same day

Variables		No antibiotic prescription	Antibiotic prescription
**Subjects n**		152,605	33,489
**Complicated LRTI (% of the population)**		3,054 (2.0)	1,018 (3.0)
**Sex[1]**	**Female (%)**	91,637 (60.0)	18,786 (56.1)
	**Male (%)**	60,967 (40.0)	14,703 (43.9)
**Age in years (% of population)**	**Median (IQR)**	55 (38 : 68)	59 (43 : 71)
	**18-49**	63,556 (41.6)	11,483 (34.3)
	**50-64**	41,794 (27.4)	9,225 (27.6)
	**65-74**	24,813 (16.3)	6,331 (18.9)
	**75-84**	15,766 (10.3)	4,214 (12.6)
	**85+**	6,676 (4.4)	2,236 (6.7)
**Estimation Comorbidities (% of population)**	**Neoplastic Disease**	15,290 (10.0)	2,960 (8.8)
	**Congestive Heart Failure**	2,076 (1.4)	578 (1.7)
	**Cerebrovascular Disease**	3,099 (2.0)	797 (2.4)
	**Diabetes mellitus**	4,118 (2.7)	1,023 (3.1)
	**Pulmonary Disease**	4,665 (3.1)	1,520 (4.5)
**Migration Background [2](% of total)**	**The Netherlands**	107,881 (70.7)	24,479 (73.1)
	**Middle & Eastern Europe**	2,734 (1.8)	645 (1.9)
	**Other Europe**	8,224 (5.4)	1,832 (5.5)
	**Turkey**	3,470 (2.3)	729 (2.2)
	**Morocco**	3,924 (2.6)	847 (2.5)
	**Suriname**	7,462 (4.9)	1,357 (4.1)
	**Dutch Caribbean**	1,749 (1.2)	292 (0.9)
	**Indonesia**	6,227 (4.1)	1,286 (3.8)
	**Other Africa**	2,332 (1.5)	479 (1.4)
	**Other Asia**	6,596 (4.3)	1,183 (3.5)
	**Other America's and Oceania**	2,005 (1.3)	358 (1.1)
**Socioeconomic status in quintiles [3]**	**1 (highest)**	35,810 (23.5)	7,751 (23.1)
	**2**	33,200 (21.8)	6,841 (20.4)
	**3**	29,876 (19.6)	6,566 (19.6)
	**4**	28,689 (18.8)	6,716 (20.1)
	**5 (lowest)**	24,239 (15.9)	5,323 (15.9)
**Current smoking (% of population)**		36,635 (24.0)	8,991 (26.9)
**Hospitalisation in the past year (% of population)**		14,720 (9.7)	2,959 (8.8)
**Clinical diagnosis of pneumonia (% of population)**		13,610 (8.9)	13,172 (39.3)
**Same-day corticosteroid prescription with pre-existing asthma or COPD exacerbation (% of population)**		45 (0.0)	105 (0.3)
**Current use of systemic corticosteroids (% of population)**		1,999 (1.3)	705 (2.1)
**Antibiotics prescribed within 30 days before GP consultation (% of population)**		6,110 (4.0)	1,264 (3.8)

1. Sex: 1 missing value 2. Migration background: 1 missing variables in the no antibiotics cohort, 2 missing in antibiotics cohort 3. SES: Socioeconomic status on household level, based on financial welfare (standardized income + standardized wealth of household) 791 (0.5%) missing in no antibiotics cohort, 292 (0.9%) missing in antibiotics cohort.

Table S2: Population Characteristics Validation dataset

Variables		No complications	Complications
**Subjects n**		25,171	585
**Sex[1]**	**Female (%)**	14,855 (59.0)	283 (48.4)
	**Male (%)**	10,318 (41.0)	300 (51.0)
**Age in years (% of population)**	**Median (IQR)**	57 (40 : 71)	74 (61 : 83)
	**18-49**	9,904 (39.4)	91 (15.6)
	**50-64**	6,397 (25.4)	85 (14.5)
	**65-74**	4,404 (17.5)	122 (20.9)
	**75-84**	3,306 (13.1)	182 (31.1)
	**85+**	1,160 (4.6)	105 (18.0)
**Estimation Comorbidities (% of population)**	**Neoplastic Disease**	3,888 (15.5)	131 (22.4)
	**Congestive Heart Failure**	463 (1.8)	52 (8.9)
	**Cerebrovascular Disease**	895 (3.6)	52 (8.9)
	**Diabetes mellitus**	1,039 (4.1)	45 (7.7)
	**Pulmonary Disease**	1,153 (4.6)	54 (9.2)
**Migration Background [2](% of total)**	**The Netherlands**	17,118 (68.0)	449 (76.8)
	**Middle & Eastern Europe**	548 (2.2)	<10
	**Other Europe**	1,441 (5.7)	26 (4.4)
	**Turkey**	674 (2.7)	15 (2.6)
	**Morocco**	752 (3.0)	13 (2.2)
	**Suriname**	1,230 (4.9)	22 (3.8)
	**Dutch Caribbean**	292 (1.2)	<10
	**Indonesia**	930 (3.7)	21 (3.6)
	**Other Africa**	462 (1.8)	<10
	**Other Asia**	1,342 (5.3)	15 (2.6)
	**Other America's and Oceania**	382 (1.5)	<10
**Socioeconomic status in quintiles [3]**	**1 (highest)**	5,670 (22.5)	89 (15.2)
	**2**	5,363 (21.3)	106 (18.1)
	**3**	5,008 (19.9)	117 (20.0)
	**4**	4,683 (18.6)	138 (23.6)
	**5 (lowest)**	4,272 (17.0)	133 (22.7)
**Current smoking (% of population)**		5,910 (23.5)	169 (28.9)
**Hospitalisation in the past year (% of population)**		2,348 (9.3)	180 (30.8)
**Working diagnosis of pneumonia (% of population)**		3,699 (14.7)	245 (41.9)
**Same-day corticosteroid prescription with pre-existing asthma or COPD exacerbation (% of population)**		35 (0.1)	<10
**Current use of systemic corticosteroids (% of population)**		456 (1.8)	47 (8.0)
**Antibiotics prescribed within 30 days before GP consultation (% of population)**		1,227 (4.9)	93 (15.9)
**Same-day antibiotics prescription (% of population)**		4,715 (18.7)	173 (29.6)

1. SES: Socioeconomic status on household level, based on financial welfare (standardized income + standardized wealth of household) 175 (0.7%) missing in no complications cohort, 2 (0.3%) missing complications cohort.

Table S3: Difference in deviance and deviance ratio between models

	Sample	Deviance	Deviance ratio
**Model 1 without SES and Migration background**	Training	0.184	0.1216
	Testing	0.191	0.1225
**Model 2 with SES and Migration background**	Training	0.184	0.1233
	Testing	0.190	0.1242
**Model 3 with SES and Migration background**	Training	0.184	0.1236
	Testing	0.190	0.1244

Table S4: Absolute risk and predicted risk of a patient's risk of developing a complicated LRTI classified into risk-groups according to NHG guidelines, stratified by SES-quintiles in derivation dataset

		Absolute risk (95% CI)	Predicted risk (95% CI)
**Risk group 1**	**1st quintile**	0.7 (0.6 – 0.8)	0.9 (0.8 – 0.9)
	**2nd quintile**	0.8 (0.7 – 0.9)	0.9 (0.9 – 0.9)
	**3rd quintile**	0.8 (0.7 – 1.0)	1.0 (1.0 – 1.0)
	**4th quintile**	0.8 (0.6 – 0.9)	1.1 (1.1 – 1.1)
	**5th quintile**	1.0 (0.9 – 1.2)	1.1 (1.1 – 1.1)
**Risk group 2**	**1st quintile**	1.9 (1.6 – 2.1)	1.7 (1.7 – 1.7)
	**2nd quintile**	1.9 (1.6 – 2.1)	1.7 (1.7 – 1.7)
	**3rd quintile**	2.2 (1.9 – 2.4)	2.1 (2.0 – 2.1)
	**4th quintile**	3.2 (2.9 – 3.5)	2.8 (2.8 – 2.9)
	**5th quintile**	2.6 (2.3 – 2.9)	2.5 (2.4 – 2.5)
**Risk group 3**	**1st quintile**	5.0 (4.5 – 5.6)	4.9 (4.8 – 5.0)
	**2nd quintile**	5.1 (4.5 – 5.7)	5.0 (4.9 – 5.2)
	**3rd quintile**	6.4 (5.8 – 7.1)	6.2 (6.0 – 6.3)
	**4th quintile**	8.7 (8.0 – 9.5)	8.9 (8.7 – 9.1)
	**5th quintile**	7.2 (6.4 – 8.0)	7.5 (7.3 – 7.7)

Table S5: Absolute risk and predicted risk of a patient's risk of developing a complicated LRTI classified into risk-groups according to NHG guidelines stratified by SES-quintiles in validation dataset

		Absolute risk (95% CI) [P-value vs 1st quintile]	Predicted risk (95% CI) [P-value vs 1st quintile]
**Risk group 1**	**1st quintile**	0.4 (0.2 – 0.6)	0.9 (0.8 – 1.0)
	**2nd quintile**	0.5 (0.2 – 0.8)	0.9 (0.8 – 1.0)
	**3rd quintile**	0.7 (0.4 – 1.0)	1.0 (0.9 – 1.1)
	**4th quintile**	0.6 (0.2 – 0.9)	1.1 (1.0 – 1.2)
	**5th quintile**	1.1 (0.6 – 1.6)	1.1 (1.0 – 1.3)
**Risk group 2**	**1st quintile**	2.0 (1.4 – 2.6)	1.9 (1.8 – 2.0)
	**2nd quintile**	2.7 (1.9 – 3.4)	2.0 (1.9 – 2.2)
	**3rd quintile**	2.3 (1.6 – 3.0)	2.3 (2.2 – 2.5)
	**4th quintile**	3.3 (2.5 – 4.0)	3.1 (3.0 – 3.2)
	**5th quintile**	3.3 (2.5 – 4.1)	3.1 (3.0 – 3.2)
**Risk group 3**	**1st quintile**	4.3 (2.9 – 5.6)	4.8 (4.6 – 5.0)
	**2nd quintile**	4.9 (3.4 – 6.4)	5.2 (5.0 – 5.4)
	**3rd quintile**	7.4 (5.6 – 9.3)	6.2 (6.0 – 6.4)
	**4th quintile**	7.8 (5.8 – 9.7)	8.2 (8.0 – 8.4)
	**5th quintile**	7.3 (5.4 – 9.2)	8.9 (8.7 – 9.1)

Table S6: Increase in mean predicted probability of a complicated course of LRTI across NHG risk strata, adjusted for all model covariates

NHG		**Marginal effect %**	**Upper boundary % 95%CI**	**Lower boundary % 95%CI**
**Risk strata 1**	**1st quintile**	REF	REF	REF
	**2nd quintile**	0.09	0.09	0.09
	**3rd quintile**	0.21	0.21	0.21
	**4th quintile**	0.28	0.28	0.28
	**5th quintile**	0.36	0.36	0.36
**Risk strata 2**	**1st quintile**	REF	REF	REF
	**2nd quintile**	0.19	0.19	0.18
	**3rd quintile**	0.45	0.46	0.45
	**4th quintile**	0.60	0.60	0.60
	**5th quintile**	0.76	0.77	0.76
**Risk strata 3**	**1st quintile**	REF	REF	REF
	**2nd quintile**	0.52	0.52	0.51
	**3rd quintile**	1.26	1.27	1.25
	**4th quintile**	1.66	1.68	1.64
	**5th quintile**	2.10	2.11	2.08

Table S7: Multivariable logistic regression sub analyses with CRP added in the model, only practices where a CRP was possible are included, prediction models refined through LASSO selection

Independent Variables	Refined full multivariable conventional model (1)<br><br>OR (95% CI)	Refined full multivariable SES model (2)<br><br>OR (95% CI)	Refined full multivariable migration model (3)<br><br>OR (95% CI)
**Sex**			
Male	**Ref**	**Ref**	**Ref**
Female	0.89 (0.82 – 0.96)	0.87 (0.81 – 0.94)	0.87 (0.81 – 0.94)
**Age**			
18 – 49	**Ref**	**Ref**	**Ref**
50 – 64	1.40 (1.23 – 1.59)	1.46 (1.28 – 1.67)	1.44 (1.27 – 1.64)
65 – 74	2.50 (2.21 – 2.84)	2.59 (2.29 – 2.94)	2.57 (2.26 – 2.91)
75 – 84	3.52 (3.09 – 4.00)	3.53 (3.10 – 4.01)	3.51 (3.08 – 4.00)
85+	6.60 (5.75 – 7.58)	6.54 (5.69 – 7.51)	6.50 (5.64 – 7.49)
**SES (quintiles where 5 is the lowest)**			
1	-	**Ref**	**Ref**
2	-	1.12 (0.99 – 1.27)	1.12 (0.99 – 1.27)
3	-	1.26 (1.12 – 1.43)	1.26 (1.11 – 1.42)
4	-	1.34 (1.19 – 1.50)	1.33 (1.18 – 1.50)
5	-	1.46 (1.29 – 1.66)	1.44 (1.27 – 1.64)
**Migration Background**			
The Netherlands	-	-	**Ref**
Middle & Eastern Europe	-	-	0.48 (0.28 – 0.82)
Other Europe	-	-	0.90 (0.75 – 1.07)
Turkey	-	-	1.16 (0.88 – 1.53)
Morocco	-	-	1.11 (0.87 – 1.43)
Suriname	-	-	1.01 (0.83 – 1.23)
Dutch Caribbean	-	-	1.16 (0.80 – 1.70)
Indonesia	-	-	1.05 (0.88 – 1.26)
Other Africa	-	-	1.12 (0.78 – 1.60)
Other Asia	-	-	0.96 (0.75 – 1.21)
Other America's and Oceania	-	-	0.92 (0.60 – 1.43)
**Comorbidities**			
Neoplastic Disease	1.60 (1.45 – 1.77)	1.62 (1.47 – 1.79)	1.62 (1.47 – 1.79)
Congestive Heart Failure	1.80 (1.54 – 2.11)	1.78 (1.52 – 2.09)	1.78 (1.52 – 2.09)
Cerebrovascular Disease	1.17 (1.00 – 1.38)	1.17 (0.99 – 1.38)	1.17 (0.99 – 1.38)
Diabetes mellitus	1.89 (1.09 – 3.29)	1.82 (1.05 – 3.17)	1.79 (1.03 – 3.11)
Pulmonary disease	1.18 (1.01 – 1.39)	1.16 (0.99 – 1.36)	1.16 (0.99 – 1.37)
**Health**			
Current smoking	1.10 (1.00 – 1.20)	1.06 (0.97 – 1.16)	1.07 (0.98 – 1.17)
Hospitalisation in the past year	2.07 (1.88 – 2.28)	2.03 (1.84 – 2.23)	2.03 (1.84 – 2.23)
Current use of oral systemic corticosteroids	2.10 (1.77 – 2.49)	2.09 (1.76 – 2.48)	2.08 (1.76 – 2.47)
Antibiotics prescribed <30 days before GP consultation	1.38 (1.20 – 1.57)	1.37 (1.20 – 1.57)	1.37 (1.20 – 1.57)
**Degree of illness**			
CRP-POC-test			
Unknown/not done	1.11 (0.57 – 2.15)	1.13 (0.58 – 2.19)	1.14 (0.59 – 2.21)
0-20	**Ref**	**Ref**	**Ref**
20-100	0.92 (0.28 – 3.02)	0.94 (0.29 – 3.10)	0.94 (0.29 – 3.``)
>100 or diagnosis of pneumonia	3.18 (1.63 – 6.19)	3.24 (1.66 – 6.32)	3.28 (1.68 – 6.38)

Same-day antibiotics prescription	1.00 (0.91 – 1.10)	1.00 (0.90 – 1.10)	1.00 (0.91 – 1.10)
Same-day corticosteroid prescription with pre-existing asthma or COPD	1.48 (0.74 – 2.97)	1.50 (0.75 – 3.01)	1.50 (0.75 – 3.01)
**Interactions**			
Age category 18-49* Diabetes Mellitus	**Ref**	**Ref**	**Ref**
Age category 50-64* Diabetes Mellitus	0.87 (0.46 – 1.64)	0.86 (0.46 – 1.62)	0.87 (0.46 – 1.64)
Age category 65-74* Diabetes Mellitus	0.67 (0.35 – 1.27)	0.66 (0.35 – 1.25)	0.67 (0.35 – 1.27)
Age category 75-84* Diabetes Mellitus	0.60 (0.31 – 1.15)	0.60 (0.31 – 1.15)	0.61 (0.31 – 1.17)
Age category >85* Diabetes Mellitus	0.33 (0.15 – 0.71)	0.34 (0.16 – 0.73)	0.35 (0.16 – 0.74)

All models were refined through LASSO selection procedures. Population includes only individuals at a GP-practice where a CRP point of care test could be conducted.

Table S8: difference in discrimination between models with CRP-POC test integrated

Derivation dataset	**Model 1 without SES and Migration background**	**Model 2 with SES**	**Model 3 with SES and Migration background**
**AUROC (95% CI)**	0.784 (0.774 – 0.793)	0.787 (0.778 – 0.796)	0.787 (0.778 – 0.797)
**ROC-curve bootstrap**	0.778 (0.769 – 0.787)	0.785 (0.777 – 0.795)	0.786 (0.778 – 0.795)
**Brier-score**	0.020	0.020	0.020
**AIC**	25045.11	25001.22	24991.92
Validation dataset	**Model 1 without SES and Migration background**	**Model 2 with SES**	**Model 3 with SES and Migration background**
**AUROC (95% CI)**	0.781 (0.764 – 0.806)	0.786 (0.770 – 0.810)	0.786 (0.770 – 0.811)
**ROC-curve bootstrap**	0.777 (0.754 – 0.798)	0.786 (0.763 – 0.806)	0.786 (0.764 – 0.807)
**Brier-score**	0.021	0.021	0.021
**AIC**	4886.68	4876.492	4875.233

Table S9: Difference in Calibration between models with CRP-POC test integrated

Derivation dataset	**Model 1 without SES and Migration background**	**Model 2 with SES**	**Model 3 with SES and Migration background**
**Intercept (95% CI)**	0.07 (-0.05 – 0.18)	0.07 (-0.05 – 0.18)	0.07 (-0.04 – 0.19)
**Slope (95% CI)**	1.020 (0.988 – 1.052)	1.020 (0.988 – 1.052)	1.022 (0.990 – 1.054)
**Brier-score**	0.020	0.020	0.020
Validation dataset	**Model 1 without SES and Migration background**	**Model 2 with SES**	**Model 3 with SES and Migration background**
**Intercept (95% CI)**	0.02 (-0.24 – 0.27)	0.00 (-0.24 – 0.25)	0.01 (-0.24 – 0.26)
**Slope (95% CI)**	1.014 (0.940 – 1.087)	1.012 (0.939 – 1.085)	1.014 (0.941 – 1.086)
**Brier-score**	0.021	0.021	0.021

Table S10: Difference in deviance and deviance ratio between models with CRP-POC test integrated

	**Sample**	**Deviance**	**Deviance ratio**
**Model 1 without SES and Migration background**	Training	0.181	0.1235
	Testing	0.191	0.1220
**Model 2 with SES and Migration background**	Training	0.181	0.1250
	Testing	0.191	0.1238
**Model 3 with SES and Migration background**	Training	0.183	0.1253
	Testing	0.190	0.1240

Table S11: Number of observations per NHG guideline and SES category strata in the derivation cohort

	SES category				
**NHG risk strata**	**1**	**2**	**3**	**4**	**5**
**1**	25,410	24,246	20,887	16,555	16,106
**2**	16,735	15,107	14,827	17,285	12,957
**3**	7,175	6,157	5,863	6,386	4,904

Figure F1: Flow diagram of data linkage process with number of observations and individuals

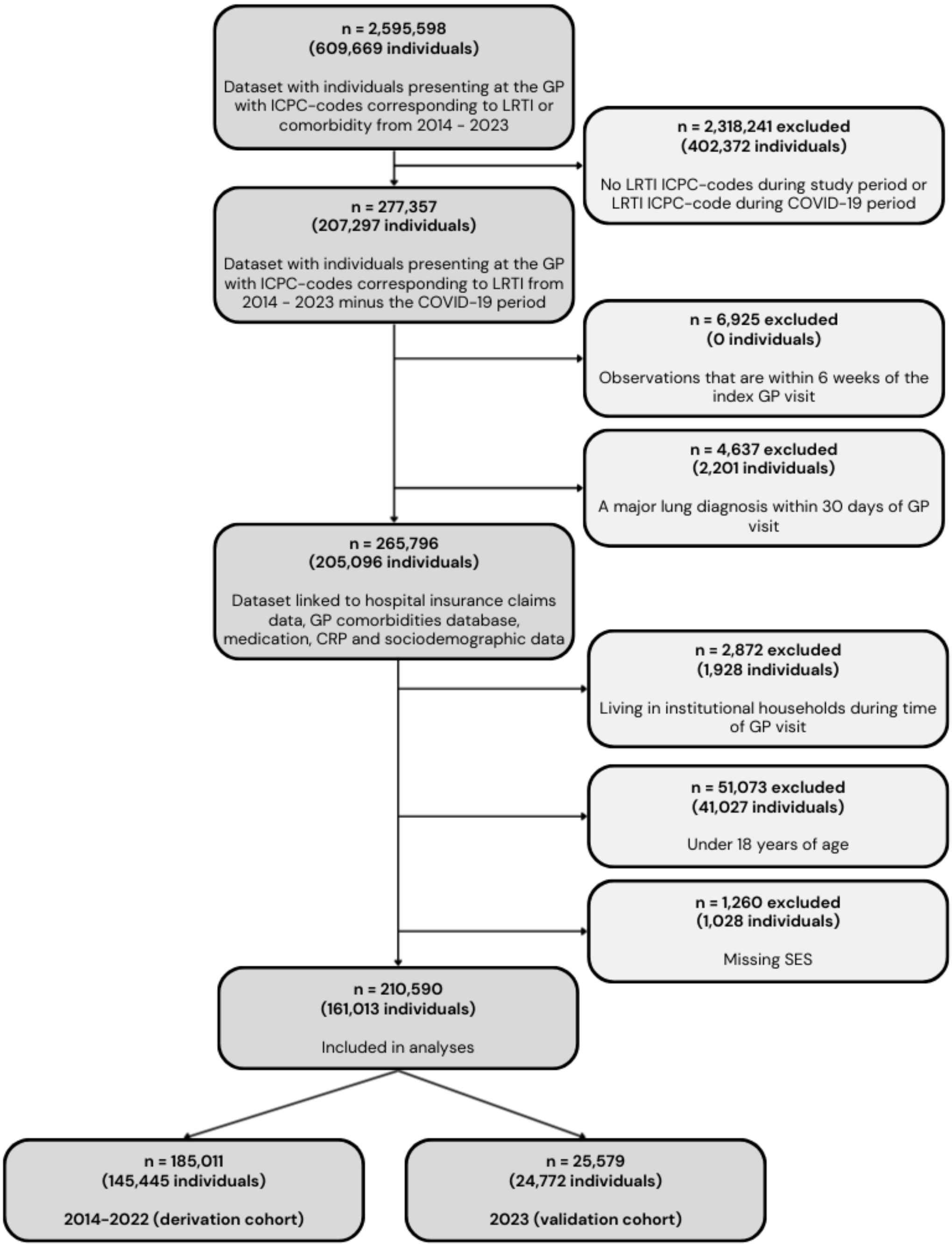

Figure F2: Calibration plot conventional model

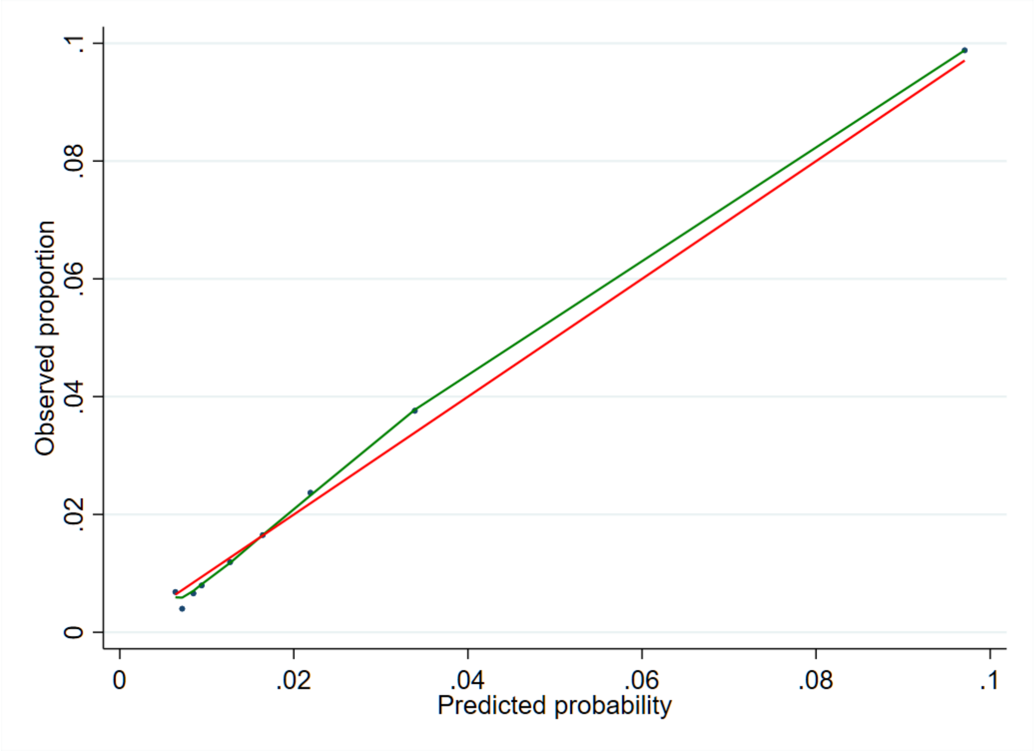

Figure F3: Calibration plot SES model

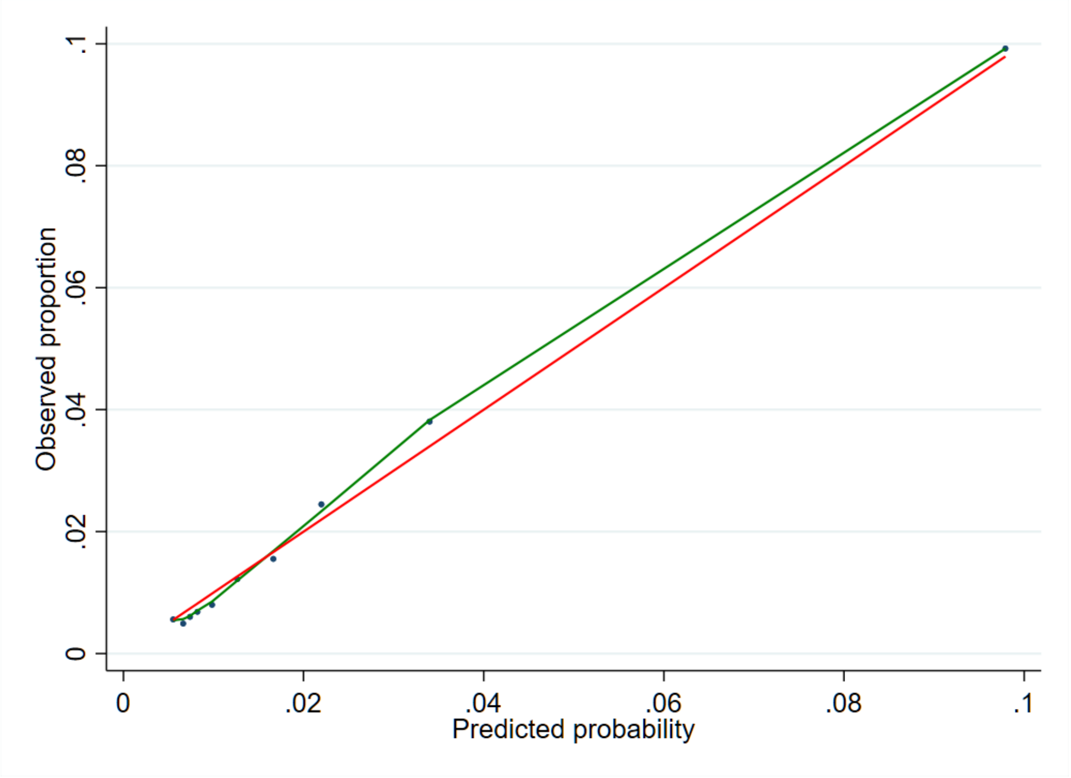

Figure F4: Calibration plot migration model

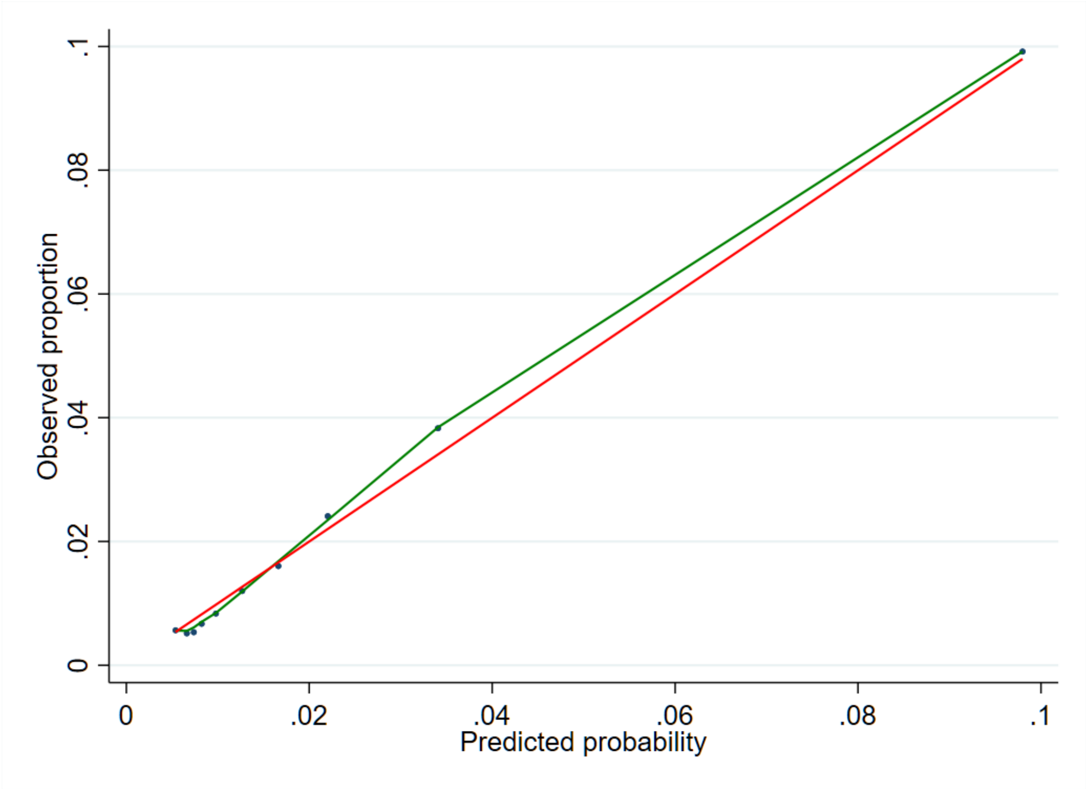

M-references

1. Statistics Netherlands. Microdata 2019. https://www.cbs.nl/en-gb/our-services/customised services-microdata/microdata-conducting-your-own-research/overview-of-all-datasets. Date last accessed: December 4 2025.
2. Fine MJ, Auble TE, Yealy DM, et al. A prediction rule to identify low-risk patients with community-acquired pneumonia. N Engl J Med. 1997;336(4):243-50.
3. World Health Organization. Anatomical Therapeutic Chemical (ATC) Classification. https://www.who.int/tools/atc-ddd-toolkit/atc-classification. Date last accessed: December 4 2025
